# Bonding Surface Designs in Fixed Orthodontic Attachments

**DOI:** 10.1155/2023/2846879

**Published:** 2023-01-23

**Authors:** Leila Sadeghalbanaei, Saeed Noorollahian, Zahra Zarei

**Affiliations:** ^1^Dental Student's Research Committee, Department of Orthodontics, School of Dentistry, Isfahan University of Medical Science, Isfahan, Iran; ^2^Department of Orthodontics, Dental Implants Research Center, Dental Research Institute, School of Dentistry, Isfahan University of Medical Science, Isfahan, Iran; ^3^Department of Orthodontics, Dental Research Center, Dental Research Institute, School of Dentistry, Isfahan University of Medical Science, Isfahan, Iran

## Abstract

Fixed orthodontic attachments/appliances work as a medium to transfer the force applied to the teeth. In bonded types, several factors affect the attachment bond strength and their clinical success. The primary approach for increasing the bond strength focused on altering the time and concentration of acid etching; however, the results showed that these changes might increase susceptibility to enamel decalcification. The bonding mechanism of orthodontic attachments may be chemical, mechanical, or a combination of both. Most attachment bonding surfaces (ABSs) have no chemical bond to resin composites. Hence, mechanical retention plays a major role. Developing more bonding surfaces by increasing the macroscopic size of the attachments has esthetic and hygienic limitations, so the ABS design plays a more important role in maintaining and improving the bond strength. In this research, different ABS designs are reviewed and categorized according to manufacturing methods and their features.

## 1. Introduction

Fixed orthodontic treatments need attachments to transfer the force applied to the target teeth [1]. In bonded types, bond strength is crucial for clinical success, and its failure delays the treatment progress and exerts an economic impact [2]. Several variables influencing the bond strength are resin-related, enamel-related, attachment-related, and bonding condition-related factors [3].

The most crucial attachment-related factor is the attachment bonding surface (ABS) morphology [2, 4–7]. The extent of contact between ABS and bonding materials, as the most important factor affecting bond strength, [8] is dependent on the ABS morphology and characteristics of bonding materials, such as viscosity and wetting ability [2, 7, 9]. Also, ABS morphology can influence the resin thickness [10], air escape, resin flow, and light penetration in light-activated bonding materials [7, 11].

Primary attempts to improve bond strength include increasing ABS macroscopic area and better ABS adaptation on the tooth surface [1, 7, 9, 12–14]. Round or triangular ABSs have less area than quadrangular ones. Trapezoid shapes have more area than rectangular, rhomboid, and square ones [4]. Extension of ABS in occluso-gingival direction in attachments with gingival offset feature is another effort to increase the bonding surface area. When the area is even, the ABS shape has no significant effect on the bond strength [15].

Aesthetic and hygienic considerations have led to smaller fixed attachments and consequently reduced macroscopic ABSs [8]. The reduced macroscopic ABS area increases the importance of its design and its microscopic area for maintaining optimum bond strength [16, 17].

The bonding mechanism of attachments may be chemical, mechanical, or a combination of both [18]. Since most ABSs have no chemical bond to resins, mechanical retention, due to ABS design, has a major role in the bonding success [5, 8, 19, 20].

Mechanical retention is achieved by producing surface roughness to increase the surface area and to develop effective undercuts for the resin to penetrate and interlock after polymerization [21].

In this paper, different ABS designs are reviewed and categorized according to manufacturing methods and their features.

## 2. Materials and Methods

Electronic databases, including PubMed, Scopus, Web of Science, and Google Scholar, were searched for relevant articles up to Jun 20, 2022. Searched MeSH terms included orthodontic fixed appliances, orthodontic brackets, and shear strength and to complete the search strategy we used other relevant terms including base design, mesh design, orthodontic attachments, pad design, bond strength, and a combination of them. Inclusion criteria were English language articles, full-text availability, and discussion on ABS-related issues. There was no time limitation. Finally, 46 articles were included in this review.

## 3. Results

The database search identified 162 articles. Of these, 14 were duplicates. Of the remaining 148, screening the titles and abstracts discarded 93. Of the remaining 55 articles, for which the full text was examined, we excluded 9. The remaining 46 articles were included in this review. The main exclusion criteria were not mentioning the type of bonding surface of the brackets used in the research and the lack of comparison between bonding surfaces as the main variable.

**Figure 1:**
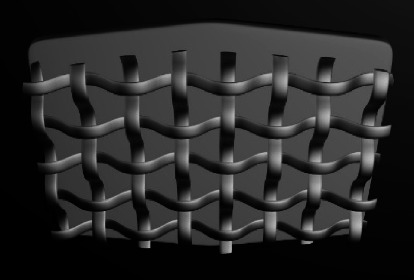
Mesh base design.

Different ABS designs have been introduced to improve mechanical retention. They can be categorized into three groups according to the method of increasing contact area and creating effective undercuts:

1. Mesh-based ABSs: Metal net/mesh is connected to an ABS. Mesh is woven fine metal strings with different sizes and holes, [20] lacing patterns, and layer numbers (Figure 1).
2. Nonmesh subtractive ABSs: Roughness, porosities, and undercuts are made by subtracting the material from ABS using laser, etchant, milling, sandblasting, etc. [11, 22].
3. Nonmesh additive ABSs: Roughness, porosities, and undercuts are made by adding metal or ceramic particles onto ABS.

### 3.1. Mesh-Based Bonding Surfaces

#### 3.1.1. Mesh Material and Size

Stainless steel No. 304 or 316L is the typical material used for the mesh fabrication [23]. The mesh gauge is determined by counting wires per linear inch in the enlarged photographs. The sizes available for single mesh designs included 40, 50, 60, 80, and 100 [24]. The size of the mesh spaces (apertures), including space area and space volume, plays a key role in the bond strength of mesh-based ABSs.[20]. The aperture diameter in the finest mesh used, i.e., 100 gauge, is about 150 microns [20, 25]. The attachments with larger apertures show better air displacement from the surface and better resin penetration into the spaces, resulting in higher bond strengths [14, 20]. Although several studies have reported that fine mesh gauges due to larger apertures demonstrate higher bond strength, some studies have indicated no significant differences between various mesh gauges [8, 13, 16, 24, 26–29].

Knox et al. revealed that certain combinations of bonding composite resin and mesh gauge could result in maximum bond strength, so determining an exact trend seemed difficult. It is related to the fact that more viscous composite resins could not penetrate into the fine spaces of some meshes [30].

#### 3.1.2. Mesh Design

The mesh string direction could be vertical or diagonal relative to the crown long axis. The diagonal pattern showed higher bond strength than the vertical one [31].

#### 3.1.3. Attachment-Mesh Connection

The mesh may be connected to the ABS by welding or brazing. The weld spots can reduce the bond strength by reducing the available retentive surface area and obliterating the mesh strings. Locating these welding points on the margins of ABS may result in voids beneath the weld spots, subsequent leakage, and enamel decalcification. The weld spots may act as stress concentration points, initiating the resin fracture at the attachment-resin interface [16, 24, 32].

To eliminate the weld spot-related problems, brazing of the mesh to the attachment was suggested. This involves joining metal parts by melting the intermediate metal fillers. The melting point of fillers should be below the solidus temperature of the metal parts and also above 840^F^ (450^C^) [33]. The brazed mesh bases have been found to show higher bond strength than the welded ones [24, 34].

#### 3.1.4. Mesh Layers (Single or Double)

The ABSs may consist of single or double mesh layers. In double-mesh attachments, the superficial one is coarser. These different types can influence the stress distribution in ABS during the debonding time, thereby causing the bracket base flexibility. In this regard, in double-mesh attachments, the stress is lower in the superficial mesh layer than in the deeper layer [20]. This reduced stress yields less stress in the cement-bracket interface due to decreased physical deformation [35]. Some studies have reported that double-mesh attachments have higher bond strength than single-mesh ones [14, 20]. And one study showed no significant difference between them [36].

### 3.2. Non-mesh Subtractive ABSs

#### 3.2.1. Perforated

**Figure 2:**
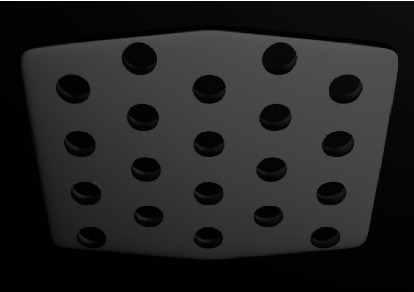
Perforated base design.

One of the earliest ABS designs was perforated, which entailed 12 to 16 holes per ABS (Figure 2). The bonding resin would seep through the perforations and interlock after polymerization to provide mechanical retention. The resin penetrating the oral environment through the perforation would increase the risk of hygienic and aesthetic problems [11, 16, 33]. These designs, especially those with peripheral perforations, showed lower bond strength than the mesh-based ones, which might be due to the smaller number of retentive components [24, 34].

#### 3.2.2. Retention Grooves

**Figure 3:**
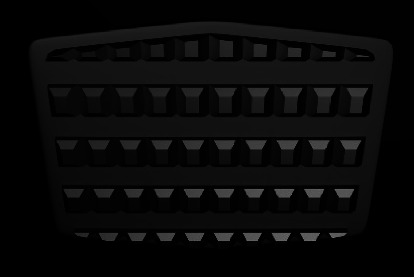
Retention grooves design.

Retention grooves are horizontal depressions on ABS which open at the mesial/distal ends (Figure 3). The *V*-shape grooves are also located vertically on ridges [19]. These vertical grooves are designed to provide an escape way for composite resins and avoid air entrapment in combination with the horizontal ones [37]. These cross-cuts increase the ABS-resin contact area, too [11]. Several studies have evaluated the bond strength of ABSs with retention grooves. Inconsistent results have been found due to different bonding agent viscosities, ABS treatment, and fabrication processes [19, 27, 30, 37–40]. Controlled studies are needed to evaluate the effect of these retentive grooves on the bond strength compared with others.

#### 3.2.3. Spherical Concavities

**Figure 4:**
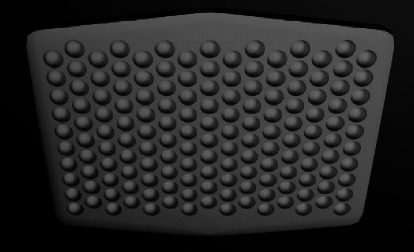
Spherical concavities design.

The shallow spherical concavities were made on ABS to provide mechanical retention (Figure 4). They are also named photo-etched, microlock, concave circular, and microetched ABSs. They have lower bond strength than mesh-based and retentive grooves [16, 24, 34, 37]. It might be due to less efficient undercuts for the resin to lock.

#### 3.2.4. Laser Structured

**Figure 5:**
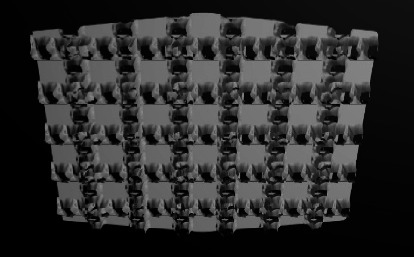
Laser structured base design.

The laser-structured ABS was introduced by Olivier Sorel in 2002 on stainless steel brackets (Discovery®, Dentaurum, Germany). In the fabrication process, the Nd: YAG laser used to produce various sizes of hole-shaped undercuts caused by selectively melted metal provided micro- and macromechanical retention (Figure 5). This type of ABS had superior bond strength to enamel and porcelain compared with the mesh-based ones [7, 41–44]. In this type of ABS, bond failure was reported at the resin-enamel interface due to maximum contact in ABS-resin interface [7].

Furthermore it seems that laser etching of the bonding surface can improve the bond strength of other ABS designs [13].

#### 3.2.5. Anchor Pylon

**Figure 6:**
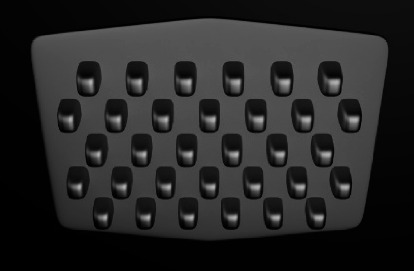
Anchor pylon design.

It consisted of miniature pylons which act as anchors penetrating and embedding in the bonding agent to provide mechanical retention (Figure 6). Three studies demonstrated better bond strength with the anchor pylon design rather than the mesh-based types [2, 45, 46].

#### 3.2.6. Concentric Grooves

**Figure 7:**
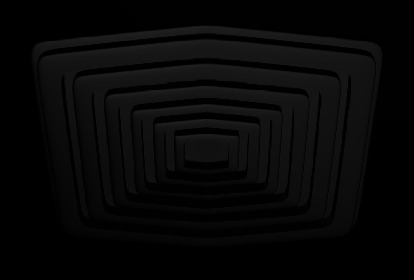
Concentric grooves design.

Concentric grooves onto ABS were introduced with two different widths (100 *μ*m and 150 *μ*m) (Figure 7). The torsional stresses in this design may transfer more uniformly to the substrate due to the hydrodynamic analogy as a physical principle and produce a higher bond strength than the mesh-based design. The wider grooves (150 *μ*m) had higher bond strength [47].

#### 3.2.7. Triangular and Quadrilateral Mesh

Attachments with triangular and quadrilateral mesh baseplates were compared by variable mesh lengths and mesh spacing diameters (mesh lengths 100, 200, and 400 *μ*m, and mesh spacing diameters 100, 150, and 200 *μ*m). The triangular mesh baseplate with a 400 *μ*m mesh length and 200 *μ*m mesh spacing had the highest bond strength, so increased aperture area has led to increased bond strength [6].

#### 3.2.8. Pad-Lock and Rail-Shaped

**Figure 8:**
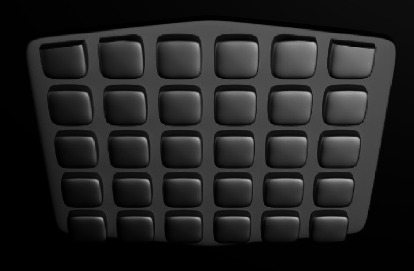
Pad lock design.

Some other innovative patterns have been introduced and mentioned in studies, including pad-lock and rail-shaped base designs. The pad-lock pattern (Figure 8) showed higher bond strength than the mesh-based design, whereas there were no significant differences between rail-shaped and mesh-based designs [17, 48].

Sandblasting, microetching, and laser etching of ABSs can improve bond strength due to increased microscopic contact area [20] although the effect of laser etching is more [13, 49].

### 3.3. Nonmesh Additive ABSs

**Figure 9:**
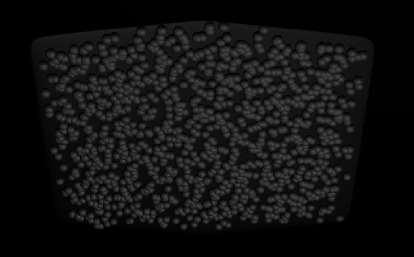
Added spherical ceramic particles.

Adding metal or ceramic particles in spherical or irregular shapes to ABSs can make roughness and efficient undercuts to provide mechanical retention (Figure 9) [11]. The metal coating processes involve sintering stainless steel or cobalt-chromium beads in various sizes onto the ABSs. Ceramic beads may fuse to the stainless steel ABSs with sintering or chemical bonding agents.

It has been revealed that porous metal-coated ABSs have higher bond strength than mesh-based ones [50, 51]. Smith and Maijer showed the sintered porous metal-coated group had the highest bond strengths and bond failure was in the resin layer, whereas the porous ceramic-coating group had the lowest result, and bond failure was in the ceramic-coating layer. As the metal particles were coarser, the bond strength was higher in this study [50].

## 4. Discussion

The clinical success of treatments done with bonded orthodontic attachments is dependent on their bond strength [26]. This bond is affected by proper enamel preparation, resin viscosity, curing and strength, and ABS morphology [3].

Numerous studies have attempted to increase the bond strength by modifying the acid etching technique, the adhesive material, and the ABS design. Although initial studies focused on altering the time and concentration of acid etching, the results showed that these changes might cause enamel loss and increase susceptibility to decalcification. Thus, a more conservative approach to increasing the bond strength is developing stronger adhesives and more retentive ABSs [23].

The bonding agent has a significant effect on the bond strength in such a way that a lower percentage of filler and a smaller particle size can lead to more penetration of the resin [32]. The variations in the thickness of adhesive material layer may affect the bond strength. Each product has its own critical thickness, in which the bond strength will be maximum [1]. However, the resin viscosity reduction to compensate for the narrow and small apertures reduces the mechanical properties of the resin and thus increases the risk of bond failure. Therefore, designs with very effective undercuts might not show the desired strength properties due to the use of resins with incompatible viscosity [30, 39].

The most important factor in ABS design which can affect the bond strength is effective contact area between resin and ABS. Debonding forces are usually parallel to ABS. Therefore, areas of ABS that are perpendicular to these forces are more effective than areas parallel to them if resin can contact them due to good penetration into undercuts. Resin penetration into undercuts is dependent on resin viscosity, dimensions of undercuts, and pressing force onto the attachment toward the tooth. The air trapped between ABS and resin inhibits resin penetration. The presence of lateral spaces from undercuts makes air escape possible, improves resin penetration, and provides an effective ABS-resin contact area [30, 39].

Several studies have investigated the influence of ABS design. Despite the common clinical perception, a smaller bracket base does not lead to insufficient bond strength if there is sufficient mechanical retention. Therefore, as the macroscopic retentive surface area of the bracket bases are reduced for aesthetic and hygienic reasons, the ABS design and morphology have a greater influence on bond strength [23].

This review reports different ABS designs and features and shows patterns with more effective undercuts, such as laser structured and anchor pylon, which have better bond strength [42, 43, 45, 46]. Also, the attachment fabrication method can influence the effectiveness of undercuts. For example, cast molded attachments have inadequate retentive grooves because the detachment of these brackets from the mold requires the absence of deep undercuts, but three-dimensional printed methods can create deeper undercuts because they are printed directly onto the base of the brackets [41].

Additionally bonding condition factors can influence bond strength, such as saliva or blood contamination. According to the previous studies these factors can cause bond failure; therefore, proper bonding technique and precise isolation during the bonding process is necessary [52, 53].

Stress distribution is another important factor affecting bond strength. The more uniform it is, the better the bond strength will be. ABSs with concentric grooves and double mesh have this advantage [30, 35, 47].

Three-dimensional printing is an emerging technology with the potential to streamline bracket production for personalized and precision orthodontics. Metal printing can be performed with both stainless steel and titanium alloys, materials commonly used in orthodontic brackets because of their biocompatibility and resistance to intraoral corrosion. Three-dimensional metal printing permits retentive features with undercuts to be printed directly onto the base of a bracket. This makes it possible to create innovative features which cannot be produced by traditional methods [54].

The limitations of the present review were in the following aspects:

1. Lack of in vivo or clinical trial studies which have investigated the effect of the bonding surface in the presence of other factors affecting the bond strength of orthodontic attachments, such as contamination with saliva or blood.
2. Limited studies which have evaluated attachment bonding surface as the main variable in orthodontic bond strength.
3. All of the studies we found in this field were in vitro studies which have several limitations, including dissimilarity to the oral environment and intraoral forces.

There is a need for further evidence-basedhigh-quality studies/randomized clinical trials (RCTs) to evaluate the effect of attachment bonding surface on bond strength and in connection with bonding condition-related factors.

3D printing technology develops possibility of designing various attachment bases and direct printing of the samples without the need for milling or casting processes. Application of this method in future studies seems to provide a new horizon.

## 5. Conclusion

As for proper enamel preparation, bonding conditions, and suitable viscosity of the bonding agent, the most important factor influencing the bond strength is the attachment bonding surface (ABS) design.

Three-dimensional designing and printing can provide effective undercuts, more air escape ways, more resin penetration, a more effective ABS-resin contact area, better stress distribution, and finally higher clinical success.

## Data Availability

Due to the review nature of our article, our data for this article are actually studies found after a wide search in databases, and upon journal request the authors can send the endnote file of all found articles separately.
